# Prevention of gross setup errors in radiotherapy with an efficient automatic patient safety system

**DOI:** 10.1120/jacmp.v14i6.4543

**Published:** 2013-11-04

**Authors:** Guanghua Yan, Kathryn Mittauer, Yin Huang, Bo Lu, Chihray Liu, Jonathan G. Li

**Affiliations:** ^1^ Department of Radiation Oncology University of Florida Gainesville FL USA

**Keywords:** patient safety, setup errors, clinic workflow, optical tracking system

## Abstract

Treatment of the wrong body part due to incorrect setup is among the leading types of errors in radiotherapy. The purpose of this paper is to report an efficient automatic patient safety system (PSS) to prevent gross setup errors. The system consists of a pair of charge‐coupled device (CCD) cameras mounted in treatment room, a single infrared reflective marker (IRRM) affixed on patient or immobilization device, and a set of in‐house developed software. Patients are CT scanned with a CT BB placed over their surface close to intended treatment site. Coordinates of the CT BB relative to treatment isocenter are used as reference for tracking. The CT BB is replaced with an IRRM before treatment starts. PSS evaluates setup accuracy by comparing real‐time IRRM position with reference position. To automate system workflow, PSS synchronizes with the record‐and‐verify (R&V) system in real time and automatically loads in reference data for patient under treatment. Special IRRMs, which can permanently stick to patient face mask or body mold throughout the course of treatment, were designed to minimize therapist's workload. Accuracy of the system was examined on an anthropomorphic phantom with a designed end‐to‐end test. Its performance was also evaluated on head and neck as well as abdominalpelvic patients using cone‐beam CT (CBCT) as standard. The PSS system achieved a seamless clinic workflow by synchronizing with the R&V system. By permanently mounting specially designed IRRMs on patient immobilization devices, therapist intervention is eliminated or minimized. Overall results showed that the PSS system has sufficient accuracy to catch gross setup errors greater than 1 cm in real time. An efficient automatic PSS with sufficient accuracy has been developed to prevent gross setup errors in radiotherapy. The system can be applied to all treatment sites for independent positioning verification. It can be an ideal complement to complex image‐guidance systems due to its advantages of continuous tracking ability, no radiation dose, and fully automated clinic workflow.

PACS number: 87.55.Qr

## I. INTRODUCTION

Safe delivery of a highly conformal dose distribution to a well‐defined target volume in radiotherapy is not an easy task. It is now becoming more complicated, due to the advent of advanced treatment techniques such as intensity‐modulated radiation therapy (IMRT) and volumetric‐modulated arc therapy (VMAT). Though a downward trend in radiotherapy incident rates has been indicated by several reports,^(^ ^1^ ^,^ ^2^ ^)^ severe incidents with detrimental effects, including death, have been reported recently and received public attention.^(3)^ Radiotherapy is a complicated multistep, multiperson process and errors can occur at any point. One of the prominent causes for radiotherapy incidents is geometric miss due to incorrect patient setup, which leads to the treatment of incorrect body parts with more than 1 cm spatial discrepancy^(^ ^3^ ^,^ ^4^ ^)^ Geometric miss can result in significant underdose to the target which can cause tumor recurrence, and overdose to healthy tissue with severe normal tissue complications. In hypofractionated radiotherapy such as stereotactic body radiotherapy (SBRT), it may result in even more severe morbidity than traditional radiotherapy.

Several reports have identified geometric miss caused by incorrect patient setup as the leading cause of radiotherapy errors. In a study of 100 radiotherapy incidents reported internationally,^(3)^ frequency of incidents due to incorrect patient setup was reported to be 21%. Excluding the 44 incidents involving brachytherapy, in which patient setup accuracy was less of an issue, this frequency would rise significantly to 37.5%.^(3)^ Clark et al.^(2)^ analyzed clinical incidents reported internally within a large academic center between 2007 and 2009, and reported that 14 out of 41 critical, major or serious incidents were geographic misses. Among the 14 incidents, one was caused by wrong target identification in treatment planning, while the other 13 incidents were due to shifting errors at patient setup. In an online report analyzing event causes for 230 misadministrations reported between 2001 and 2009 in New York from New York State Department of Health, incorrect body part treated was the leading type of errors at 46%, most often due to incorrect patient setup.^(5)^

The challenge in patient setup is to accurately localize the patient to the same treatment position as planned in each treatment session. The past decade has seen rapid expansion in technological tools to facilitate accurate patient localization. Examples include immobilization devices with couch indexing ability and image‐guided radiotherapy (IGRT). Indexed immobilization devices not only ensure repeatable patient fixation, but also reduce patient setup errors by providing initial approximate target localization. X‐ray‐based image‐guided technologies, such as cone‐beam CT (CBCT), enable the visualization of internal anatomy with sufficient soft tissue contrast. It allows corrections for misalignment or interfraction motion through registration with reference CT images.^(6)^ IGRT systems capable of continuous tracking such as AlignRT (Vision RT Ltd., London, UK), C‐Rad Sentinel (C‐Rad AB, Uppsala, Sweden), SonArray (Varian Medical Systems, Palo Alto, CA), and ExacTrac Optical‐Tracking System (BrainLAB, Heimstetten, Germany) have also been developed for the purpose of patient setup guidance. These systems have the potential to eliminate patient setup errors and significantly reduce setup uncertainty, as evidenced by many studies. Bissonnette and Medlam^(1)^ reported a 50% overall decrease in the rate of incidents caused by localization errors after wide‐spread introduction of IGRT on six of their 16 linear accelerators. Clark et al.^(2)^ pointed out that none of the 13 geometric misses occurred on treatment units equipped with daily image guidance system.

However, employment of these advanced technological equipment and tools does not guarantee that radiotherapy is immune to setup errors.^(^ ^7^ ^,^ ^8^ ^)^ Patient setup, a process which cannot be automated, is subject to human errors. There are several contributing factors. First, sufficient formal training is not always provided to personnel who operate the devices and interpret the results. Incorrect interpretation leads to incorrect adjustment of treatment position and, therefore, to setup errors. For example, a great risk in treating thoracic spine is the treatment of the wrong vertebral body.^(9)^ Due to similarities in the bony structure in this region, incorrect alignment could occur as a result of misregistration using either orthogonal imaging or CBCT. Second, as complexity of the tools increases, complexity of control over the devices and workload for therapists increases significantly as well, as evidenced by the increasing number of computer monitors in control rooms.^(8)^ Therapists could lose attention to correctness of treatment delivery when streamlined workflow and standardized control is lacking. Third, while most IGRT devices have great geometric precision,^(10)^ they come with limitations which could impact their ability in ensuring patient safety. For example, widely used radiographic system such as CBCT doesn't track patient position change and, thus, only represents patient position at the time of image acquisition. After CBCT imaging, treatment couch could be accidently moved for particular reasons (e.g., clearance check), and not moved back to intended treatment position. Modern delivery system has interlocks to detect such errors, but also provides user flexibility to override them. If the interlock is negligently overridden, patient can be treated at completely wrong site. In addition, CBCT (and most radiographic‐based IGRT systems) cannot be performed for noncoplanar setups, meaning that imaging position would differ from treatment position if noncoplanar treatment beams are used.^(11)^ As another example, continuous tracking systems such as ExacTrac Optical‐Tracking System, AlignRT, and C‐Rad Sentinel, are usually employed in the treatment of limited disease sites in current practice, for various reasons. Therapists on machines equipped with multiple IGRT devices often find themselves switching between devices from one patient to another, depending on the treatment site of each patient. Fourth and most importantly, immediate and independent position verification is not always available to the therapists to show that the patient has been positioned exactly as planned, especially when the above‐mentioned limitations of the employed IGRT systems are encountered.^(8)^

In this paper, we report an efficient, automatic patient safety system (PSS) which addresses issues contributing to gross setup errors and safeguards patient treatment. The system utilizes CCD cameras to track a single infrared reflective marker (IRRM) affixed on patient skin or immobilization device. It is a general‐purpose system, applicable for all disease sites. The system provides continuous and independent verification on patient setup accuracy. With a fully automated workflow, the PSS can be an ideal complement to complicated IGRT systems to ensure patient safety in radiotherapy.

## II. MATERIALS AND METHODS

### A. System overview

**Figure 1:**
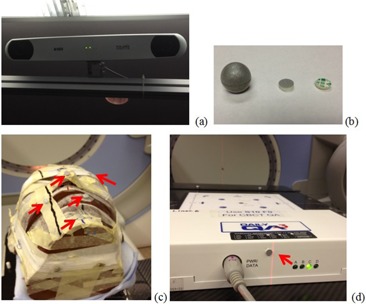
A NDI Polaris CCD camera (a) mounted to treatment room ceiling, (b) commercial spherical IRRM and home designed flat‐surfaced IRRM (front and back), (c) an anthropomorphic head phantom with five flat‐surface IRRMs (indicated by arrows) for PSS accuracy test. One IRRM was used each time in the test with the other four blocked. A flat‐surfaced IRRM (d) mounted on a daily QA device for PSS morning QA.

The system consists of a pair of Polaris CCD cameras (Northern Digital Inc., Waterloo, Ontario) mounted on the treatment room ceiling (see Fig. 1(a)), a single IRRM affixed on patient skin or immobilization device, and a set of in‐house developed software. The software communicates with the control box of the cameras through a regular serial cable and a proprietary data cable. An internal processing unit conducts a process called triangulation to determine the coordinates of IRRMs in its native coordinate system. The three‐dimensional (3D) coordinates of up to 50 IRRMs can be sent back to the computer at the same time. System calibration, workflow, and daily quality assurance (QA) procedure are described in the following sections.

### B. System calibration

A calibration procedure was developed to convert the camera native coordinate system to absolute room coordinate system. A calibration jig consisting of five commercial IRRMs (Fig. 1(b)) was used for this purpose. The coordinates of each IRRM relative to the center of the jig were determined via CT scan and collected in a 5×3 matrix, A. Under the guidance of CBCT, the jig was then placed on the treatment couch with its center aligned with imaging isocenter. Congruence of imaging isocenter and machine isocenter was confirmed with a ball bearing phantom.^(6)^ The coordinates of the IRRMs were sampled by the cameras and collected into another matrix, B. A rotation matrix, R, and a translation (shift) matrix, S, satisfying

(1) A=BR+S

were solved by minimizing the following expression:

(2) $$\varepsilon^{2}=\left\|A-\text{BR}-S\right\|$$

This is known as the relative pose problem in computer vision and has been studied extensively.^12^, ^13^, ^14^ In this work, we employed a general analytical solution via singular value decomposition (SVD),^(14)^ and the steps were summarized as following:

1. The centroids ($A_{c}$) and ($B_{c}$) were subtracted from A and B, respectively, to align the origins of the two coordinate systems.
2. The SVD of $B^{T}$ A was computed as

(3) $$B^{T}A={\text{UWV}}^{T}$$

where *U* and *V* are unitary matrices, and *W* is a diagonal matrix whose diagonal entries are equal to the singular values of $B^{T}$ A.

3. The solutions of R and S were given as

(4) $$\begin{array}{l} R=U\left(\begin{array}{lll} 1 & 0 & 0\\ 0 & 1 & 0\\ 0 & 0 & \text{det}(UV^{T}) \end{array}\right)V^{T}\\ \;\;\;\;\;\;\;\;\;\;\;\;\text{and},\\ S=A_{c}-B_{c}R \end{array}$$

### C System workflow

**Figure 2:**
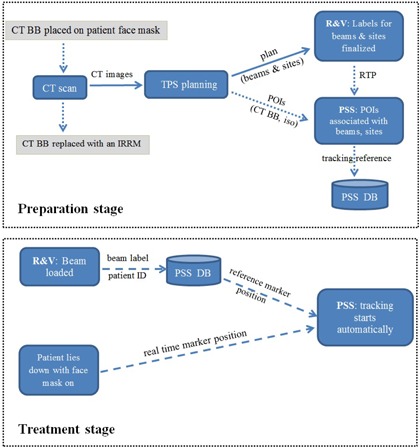
Workflow of the PSS for head and neck patient. In preparation stage, reference tracking information is created by associating POIs exported from TPS with treatment beams and sites exported from R&V. Dotted arrows represent work or data flow introduced by PSS. In treatment stage, reference tracking information retrieval is triggered by beam loading in R&V. Dashed arrows represent data flow introduced by PSS and completed without user intervention.

Great emphasis was placed on developing a smooth clinical workflow for the system. The workload added to the therapists should be kept to minimum, which was critical for the system to be adopted into clinic use. Minimal workload would also help minimize the possibilities of human errors. Workflow of the PSS is described in the following sections as preparation stage and treatment stage. Figure 2 shows a demonstrative workflow of the system when used on head and neck (H&N) patients treated with aquaplastic face masks.

#### C.1 Preparation stage

Patient preparation includes CT simulation and information transfer from the treatment planning system (TPS) (${\text{Pinnacle}}^{3}$; Philips Radiation Oncology Systems, Fitchburg, WI) to PSS. Prior to CT simulation, a CT BB is placed on patient surface at a location close to intended treatment area to ensure that it is included in the scan. After the scan, the BB is removed and its location is marked with a skin tattoo. The CT BB is identified as a point of interest (POI) in TPS and its coordinates are sent to a designated network folder along with each treatment isocenter. The approved treatment plan in TPS is transferred to the record and verify (R&V) system (MOSAIQ; Elekta Oncology Systems Ltd., Crawley, UK), where treatment beams and treatment sites are appropriately labeled and finalized before physics approval. The approved plan (including treatment beams and their associated sites) is then exported from the R&V in radiation treatment plan (RTP) format to the same network folder. The data import module of PSS associates each treatment site in the RTP file with POIs exported from TPS, including treatment isocenter and the CT BB. The CT BB coordinates relative to the respective isocenter of each treatment site predict the room coordinates of the CT BB, which will be replaced with an IRRM at treatment. All treatment beams and their associated treatment sites in the RTP file are imported into PSS database (DB) to facilitate workflow automation at treatment stage, as described below.

#### B.2 Treatment stage

At our institution, external‐beam radiotherapy is performed on dual energy linear accelerators (Synergy; Elekta). The R&V systems for these machines are configured to create an external file when a treatment beam is loaded. The external file contains patient identification and label of the currently loaded beam. PSS uses the beam label to retrieve the associated treatment site, and in turn, the reference tracking data from its database. Patient information loading in PSS is thus fully automated, and no therapist intervention is necessary. When setting up patient for treatment, therapists will locate the skin tattoo indicating the CT BB position and place a specially designed disposable flat‐surface IRRM (see Fig. 1(b)). The IRRM is fabricated by covering one side of a piece of round double‐sided sticky tape (6 mm diameter and 1 mm height) with reflective tape. The other side is protected by peelable sheet which makes mounting of the IRRM easy. PSS uses the IRRM as surrogate to verify patient setup. The discrepancy (in three dimensions) between its room position and reference position is continuously monitored and displayed on a computer monitor inside the treatment room. The same information is displayed on another computer monitor in the control area. If the discrepancy exceeds prespecified thresholds, the therapists can take appropriate corrective action with assistance of a physicist.

**Figure 3:**
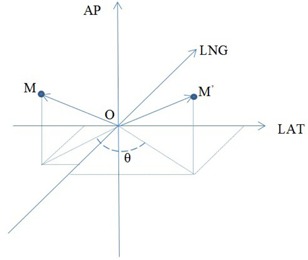
For a noncoplanar treatment beam (with couch rotation 0), the vector OM connecting isocenter and the IRRM is rotated around AP axis in the same way (direction and angle) as couch rotates. Coordinates of new IRRM position M’ are used as reference.

To cope with noncoplanar treatment, the vector connecting isocenter and IRRM is rotated around the vertical axis in the same way (direction and angle) as couch rotates to calculate reference location of the IRRM (see Fig. 3).

#### C.3 Workflow automation

The above described workflow requires the therapist to locate the skin tattoo and place an IRRM in each treatment session. To repeat this for each patient could be a significant burden to the therapist and a potential source of error since the IRRM could be placed at wrong locations. Likelihood of such errors increases if the patient had already gone through multiple radiotherapy courses, which could leave multiple skin tattoos on the patient.

**Figure 4:**
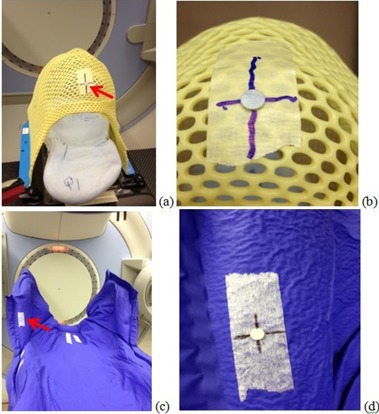
An aquaplastic face mask (a) with a flat‐surface IRRM (indicated by arrow); (b) a closer look of IRRM on the face mask; (c) a half‐body mold with a flat‐surface IRRM; (d) a closer look of the IRRM on the body mold.

To minimize the workload and avoid potential errors, the IRRM can be permanently mounted on patient immobilization device. This is easy to achieve for patients treated with aquaplastic face masks (for brain or H&N disease). The CT BB can be directly affixed on the face mask in CT scan and replaced with an IRRM afterwards. Its location is selected to be over patient chin area which ensures its visibility to both cameras in the treatment room (see Figs. 4(a) and (b)). With this approach, the IRRM is readily available before treatment starts. System workflow is fully automated and no intervention is needed from the therapist in the treatment room. Once a beam (typically setup beam) is loaded in R&V and the face mask is placed on the patient, PSS automatically starts to track the IRRM continuously and evaluates setup accuracy in real time.

The same approach can be applied for patients treated with body mold (for patients with disease not in the brain or H&N area). For patients with disease in their extremities, a spot on the body mold close to the disease can be conveniently selected to place a CT BB. But for patients with disease in abdominalpelvic area, it can be problematic. At the time of CT simulation, it may be difficult to locate a spot on the body mold that simultaneously guarantees (1) the visibility of CT BB in CT images, (2) the visibility of the IRRM (replacing CT BB after the scan) to both cameras, and (3) that the IRRM will not be easily scraped off when the patient gets in and out of the mold for treatment. For these reasons, an alternative workflow was adopted in these cases. The CT BB is placed on patient middle anterior surface during CT scan and marked with a skin tattoo afterwards. In the first treatment session, therapists will locate the tattoo and temporarily place an IRRM for initial verification. The IRRM can be installed on a small plastic plate for easy handling. Once the verification is successful, a convenient location on the body mold can be selected to permanently mount an IRRM. Without concern of CT scan range, it is relatively easy to pick an ideal spot on the body mold. The selected location ensures visibility of the IRRM to the cameras and it will not be easy for the patient to scrape off the IRRM (see Figs.4(c) and (d)). The new location of the IRRM is then captured as reference for future treatment sessions. No therapist intervention is needed after the first treatment session.

### D. Daily quality assurance

A daily QA procedure is necessary to ensure the accuracy and consistency of the system. We have developed a simple and efficient daily QA procedure which can be integrated with other daily QA activities, the majority of which are performed with a daily QA device (Daily QA3, Sun Nuclear Corp., Melbourne, FL).^(15)^ At the time of initial system calibration, an IRRM is permanently mounted on the end side of the daily QA device (see Fig. 1(d)), facing the cameras. After aligning the daily QA device to the room isocenter with the CBCT system, reference position of the IRRM is captured and associated with a daily QA beam in R&V. As a result of daily IGRT QA, the daily QA device is aligned to room isocenter with CBCT guidance.^(15)^ When the daily QA beam is loaded in R&V, consistency of PSS indicated by position variation of the IRRM is immediately displayed.

### E. System accuracy evaluation

#### E.1 System stability

Short term stability of the system was evaluated by monitoring the IRRM mounted on a daily QA device for a consecutive 10 minutes. The Polaris cameras were sufficiently warmed up before the study and the daily QA device was kept stationary on the couch. Real‐time position of the IRRM was captured, approximately 15 frames per second with the cameras, and recorded in a log file for analysis. Variations of IRRM position in the 10‐minute period in each orthogonal direction were used to evaluate short term stability of the system. Long‐term stability of the system was also evaluated with the daily QA device, using the same setup for daily QA. The device was aligned to room isocenter with CBCT guidance once a week over a 12‐week period. Long‐term stability of the system was assessed with position variations of the IRRM in these tests.

#### E.2 Phantom study

Accuracy of the PSS was first evaluated with an anthropomorphic head phantom in an end‐to‐end test, as shown in Fig. 1(c). The head phantom was scanned on a multislice CT scanner (Brilliance; Philips Medical Systems, Cleveland, OH). To investigate the effect of different CT BB locations, five CT BBs were affixed on the phantom for CT scan. One CT BB was located right over the chin area and the other four were at random locations on the phantom surface. All CT BBs were replaced with IRRMs after the scan. An isocenter was arbitrarily defined inside the head phantom in TPS. Its coordinates were then sent to PSS, along with those of the CT BBs. In treatment room, the head phantom was positioned to treatment isocenter under guidance of CBCT. PSS used one IRRM at a time, with the other four IRRMs blocked from the camera, to verify setup accuracy.

To evaluate the ability of the system in detecting couch shifts of various magnitudes, the phantom was positioned to 30 different locations by shifting the couch ±0.5,±1,±2,±5or±10cm along each axis. The PSS‐observed couch shifts were compared to nominal couch shifts. The IRRM over the chin area was used in this study. Its room coordinates before couch shifts were captured as baseline. This experiment was repeated with a 60° couch rotation to evaluate system performance under noncoplanar treatment conditions.

#### E.3 Patient study: head and neck patients

PSS has been implemented in four treatment rooms at our institution since April 2012. Setup of all patients in each treatment session has been verified with the system. Twenty patients with disease in the H&N area were included in this retrospective study to evaluate system accuracy. The patients were immobilized with an H&N immobilization system (AccuFix; Q‐Fix, Avondale, PA). The indexed immobilization system related CT BBs embedded in CT couch top to treatment isocenter through indexing bars. This couch indexing system (CIS) provided initial target localization, which was subsequently refined with in‐room CBCT guidance. PSS started automatic position verification at the moment the face mask was placed on patient. Position deviations of the IRRM, observed with PSS before and after CBCT‐guided couch adjustment, were used to evaluate system accuracy against CIS and CBCT system, respectively. Differences between PSS‐observed couch shifts and couch shifts from CBCT registration were also reported.

#### E.4 Patient study: abdominalpelvic patients

For patients with disease in the thoracic or abdominalpelvic region, the IRRM would be placed directly on patient chest or abdomen in the first treatment session. With the automated workflow, the IRRM would be transferred to patient immobilization device. However, if it is desirable to continuously monitor the patient during treatment, or when body mold is not used in patient treatment (e.g., for palliative purpose), the IRRM could be affixed on patient skin in each session. Real‐time position of the IRRM is subject to breathing motion when directly placed on patient chest or abdomen. Maximum range of external surface motion can be over 20 mm depending on factors such as patient health condition and breathing pattern, which directly impacts action level selection in PSS. To evaluate the effect of breathing motion, twenty patients with disease in the abdominalpelvic region were included in this study. The IRRM was placed directly over the abdominal area where breathing motion mostly occurred. Motion range throughout the entire treatment session and accuracy of the system when compared to CIS and CBCT systems were reported for these patients.

## III. RESULTS

### A. System workflow efficiency

The expected total time in patient preparation for the use of the PSS is less than 5 minutes. For brain, H&N, and extremity patients, the CT BB can be placed on immobilization devices and replaced with an IRRM after CT scan, and no skin tattoo is needed. For abdominalpelvic patients, the CT BB has to be affixed on patient skin, thus requiring a skin tattoo. In our current practice, two sets of skin tattoos are made for each abdominalpelvic patient to assist patient setup (mainly body rotation correction). Each set includes one anterior and two side tattoos, with one on each side. Either of the two anterior tattoos, when included in CT scan, can be selected to affix the CT BB. Therefore, no extra tattoo needs to be made for the use of PSS. Information transfer from TPS to PSS, implemented with scripting language and standard FTP (file transfer protocol), takes a single button click in TPS. An efficient software module is implemented to combine information from TPS and R&V and import into the PSS database.

At treatment stage, if an IRRM has been mounted on immobilization devices, no therapist intervention is needed. Otherwise, therapists will locate the selected anterior skin tattoo for initial verification, select a location on immobilization device to permanently mount an IRRM, and capture the new location with the PSS. This procedure adds approximately 2 to 3 minutes to patient setup time in the first treatment session. No intervention is needed in future treatment sessions.

### B. System stability

The results of short‐term stability test are shown in Table 1 (line A). The standard deviation of the position variation in a 10‐minute period was close to 0 in all directions. Maximum variation was 0.3 mm along the longitudinal direction. This variation was in part due to vibrations in the ceiling.^(16)^ Table 1 (line B) shows the results of long term stability test, in which the IRRM was positioned to the same location weekly along with the daily QA device under CBCT guidance. An evenly distributed deviation was observed along the three axes with mean errors of 0.5±0.4mm,0.5±0.5mmand0.6±0.5mm for lateral, longitudinal, and anteroposterior direction, respectively. The combined 3D deviation was 1.0±0.6mm on average, with a maximum of 2.0 mm. The deviation included uncertainty from CBCT image registration, as well as automatic couch movement. The accuracy of both the CBCT image registration^(^ ^17^ ^,^ ^18^ ^)^ and automatic couch movement^(19)^ was reported to be on the order of 1.0 mm.

**Table 1:** System stability: Line A ‐ short term stability as evaluated by observing position variation of a stationary IRRM during a 10‐minute span (9000 samples); Line B ‐ long‐term stability during a 12‐week span as evaluated by observing weekly position variation of the IRRM mounted on a daily QA device which was positioned to the same location under CBCT guidance

		*ΔLAT (min)*	*ΔLNG (min)*	*ΔAP (min)*	*ΔVECT (min)*
A.N=9000	SD	±0.0	±0.1	±0.1	±0.1
	max. (abs)	0.2	0.3	0.2	0.4
B.N=12	mean±SD	0.5±0.4	0.5±0.5	0.6±0.5	1.0±0.6
	max. (abs)	1.3	1.6	1.5	2.0

### C. System accuracy: phantom study

The results of the phantom study are shown in Table 2. In the end‐to‐end test with five individual IRRMs, mean error on all three axes was within 1.5 mm; the maximum deviation was 2.3 mm for both longitudinal and anteroposterior axes (Table 2 line A). The combined 3D deviation was 2.2±0.8mm, on average, with a maximum of 3.1 mm. Variation (less than 2.3 mm on all three axes) was observed among individual IRRMs mounted at different locations. The IRRM situated over chin area was selected in the remaining studies due to its superior visibility. In the end‐to‐end test, deviation with this marker was 0.8 mm, 0.6 mm, and 0.1 mm along lateral, longitudinal, and anteroposterior axis, respectively.

Table 2 line B shows the difference between nominal couch shifts and PSS observed shifts. In this test, 30 combinations of shifts between 0.5 cm and 10 cm were applied to the couch along each axis. Maximum difference on individual axis was less than 1.5 mm and maximum combined 3D difference was less than 2.0 mm. When there was a 60° couch rotation, maximum difference on individual axis and combined 3D difference increased to 1.7 mm (along anteroposterior axis) and 2.8 mm, respectively, as shown in Table 2 line C. A possible reason for the increased deviation with couch rotation is that the PSS tracks IRRM instead of the treatment isocenter. Small uncertainty in couch rotation can translate into large spatial deviation, depending on the distance between the IRRM and treatment isocenter (see Fig. 3). For example, a couch rotation error of 1° (typical uncertainty of couch rotation) results in an effective spatial displacement of 1.7 mm when the IRRM is 10 cm away from the isocenter.

**Table 2:** System accuracy evaluated with an anthropomorphic head phantom. Shown are: system deviations (mean±standard deviation) (Line A) for the end‐to‐end test with individual IRRMs; the test (Line B) with couch shifts along each axis by ±0.5,±1,±2,±5or±10cm; the same test (Line C) as in Line B except with a 60° couch rotation

		*ΔLAT (min)*	*ΔLNG (min)*	*ΔAP (min)*	*ΔVECT (min)*
A.N=5	mean±SD	1.1±0.7	1.4±0.8	1.0±0.8	2.2±0.8
	max. (abs)	2.0	2.3	2.3	3.1
B.N=30	mean±SD	0.3±0.2	0.5±0.4	0.4±0.3	0.7±0.2
	max. (abs)	0.5	1.2	0.9	1.6
C.N=30	mean±SD	0.3±0.3	0.5±0.5	0.6±0.6	0.9±0.7
	max. (abs)	0.8	1.5	1.7	2.8

### D. System accuracy: head and neck patients

The results of system accuracy evaluated retrospectively on 20 head and neck patients are shown in Table 3. Table 3 lines A and B show deviations of the PSS with respect to the CIS and CBCT systems, respectively. Results in the table were obtained by analyzing recorded real‐time marker positions before and after CBCT‐guided automatic couch shift. Mean deviation of the system along each axis was less than 2.5 mm with respect to both systems, but the maximum deviation reached 3.8 mm (lateral direction) when compared with CIS, and 5.5 mm (anteroposterior direction) when compared with CBCT system. The maximum combined 3D deviation of the system was less than 8.0 mm when compared to both systems. The system showed better agreement with CIS than with CBCT system. One of the reasons is that, unlike CBCT system, PSS and CIS do not account for daily setup variation, as well as patient internal anatomy change. For this reason, CBCT system represents the desired treatment position and is used to guide treatment. Table 3 lines C and D show intended couch shifts based on CBCT image registration and residual errors as observed with PSS, respectively. Intended couch shifts were evenly distributed among three axes, with a mean between 2.4 mm and 3.5 mm and a maximum between 5.6 mm and 7.0 mm. Combined 3D shift had an average of 5.8±2.1mm, with a maximum of 9.0 mm. Residual errors, observed with PSS, had an average less than 1.0 mm and a maximum less than 2.0 mm along all three axes. The maximum combined 3D residual error was less than 2.0 mm. These results show the ability of the PSS in (1) predicting patient treatment position with sufficient accuracy, and (2) accurately tracking automatic couch motion, which lacks independent verification in current practice.^(19)^

**Table 3:** System accuracy retrospectively evaluated on 20 head and neck patients. Shown in the table are: deviations with respect to CIS (Line A); deviations with respect to CBCT system (Line B); couch shifts determined by CBCT system (Line C); residual errors of automatic couch motion observed by PSS (Line D)

N=20		*ΔLAT (min)*	*ΔLNG (min)*	*ΔAP (min)*	*ΔVECT (min)*
A.	mean±SD	1.7±1.0	1.8±0.9	1.3±0.9	3.9±1.1
	max. (abs)	3.8	3.3	3.1	4.8
B.	mean±SD	2.4±1.2	2.3±1.7	2.1±1.5	4.3±1.5
	max. (abs)	5.2	4.8	5.5	7.6
C.	mean±SD	2.2±1.8	2.8±1.8	3.5±1.9	5.8±2.1
	max. (abs)	6.5	5.6	7.0	9.0
D.	mean±SD	0.6±0.3	1.0±0.7	0.5±0.3	1.5±0.4
	max. (abs)	1.0	1.5	1.9	1.9

### E. System accuracy ‐ abdominalpelvic patients

Table 4 shows the results for abdominalpelvic patients. The IRRM was placed directly on patient abdominal area in this study. Due to significant breathing motion, average IRRM position of at least four breathing cycles (~20seconds) was used in the analysis. When compared with CIS, average deviations were between 2.8 mm and 3.9 mm along all three axes, as shown in Table 4 line A. The maximum deviations were less than 6.0 mm for each axis. Average deviations along each axis, in general, were approximately 1 to 2 mm larger for these patients than for head and neck patients. While breathing motion partially contributed to these larger deviations, system accuracy for these patients was mainly limited by the accuracy in placing the IRRM on moving patient surface. Additional uncertainty of approximately 1 to 2 mm could have been introduced when placing the 6 mm diameter IRRM over the tattoo on patient abdominal, compared to placing an IRRM on stationary face mask. Situated on loose skin, relative position of the tattoo with respect to treatment isocenter could vary from day to day, and from the time of CT simulation. Comparison of PSS with CBCT system yielded similar results as shown in Table 4 line B, except the maximum deviation along longitudinal direction, which was 4 mm larger. Table 4 line C shows the intended couch shifts resulting from CBCT registration. These results were comparable to head and neck patients along lateral and anteroposterior directions (Table 3 line C), but twice as large along longitudinal direction, with an average of 4.5 mm and a maximum of 12.5 mm. Average residual errors of couch shift observed with PSS, were below 2.0 mm for all three axes, as displayed in Table 4 line D. A maximum difference of 3.7 mm was observed along anteroposterior direction. Table 4 line E shows the range of patient motion, indicated by the motion of the IRRM. While the motion along either lateral or longitudinal direction was no more than 6.0 mm, average motion of 9.7±3.3mm was observed along anteroposterior direction, with the maximum being 16.2 mm. The combined 3D motion had a maximum of 17.2 mm for these patients.

**Table 4:** System accuracy retrospectively evaluated on 20 abdominalpelvic patients. Shown in the table are: deviations with respect to CIS (Line A); deviations with respect to CBCT system (Line B); couch shifts determined by CBCT system (Line C); residual errors of automatic couch motion observed by PSS (Line D); patient motion range observed by PSS (Line E)

N=20		*ΔLAT (min)*	*ΔLNG (min)*	*ΔAP (min)*	*ΔVECT (min)*
A.	mean±SD	2.8±1.6	3.9±1.6	3.4±1.8	6.3±1.3
	max. (abs)	4.9	5.9	6.0	8.1
B.	mean±SD	4.1±2.2	4.5±2.4	2.5±2.2	7.9±1.9
	max. (abs)	6.5	9.9	6.2	11.9
C.	mean±SD	3.3±2.3	4.5±4.0	2.1±1.8	7.5±3.6
	max. (abs)	6.4	12.5	6.2	13.5
D.	mean±SD	1.0±0.8	1.3±0.6	1.8±1.1	2.8±1.2
	max. (abs)	2.1	2.4	3.7	4.1
E.	mean±SD	2.0±1.2	3.8±1.5	9.7±3.3	9.8±3.9
	max. (abs)	3.5	5.9	16.2	17.2

## IV. DISCUSSION

While overall incident rate in radiotherapy has decreased significantly due to rapid development in technology tools, severe incidents with detrimental effects have been reported in recent scientific publications as well as the public press. Radiotherapy is a complicated multistep process with human involvement in each step. Patient setup on treatment couch, a critical step which cannot be automated, is subject to human errors, even with the employment of advanced IGRT systems. The purpose of this work is to develop an efficient, automatic, and general‐purpose PSS to prevent gross setup errors with immediate and independent patient position verification.

### A. System accuracy for gross error detection

Our results demonstrate that the system has adequate accuracy in detecting gross setup errors. The system displayed good reproducibility, with short‐term and long‐term variation within 0.5 mm and 2.0 mm, respectively. The end‐to‐end test with an anthropomorphic head phantom revealed system accuracy on the order of approximately 1 to 2 mm when compared with CBCT. It accurately tracked couch motions along each axis to within 1.2 mm when there was no couch rotation, and within 1.7 mm when there was 60° couch rotation. These results show that the system is able to accurately predict patient treatment position and detect unintended couch shifts. On head and neck patients, system tracking accuracy slightly decreased due to finite CT slice thickness (3 mm), which affected POI identification of CT BB in TPS and accuracy of IRRM placement on real patient. However, the accuracy was generally within 6.0 mm in each orthogonal direction when compared to both CIS and CBCT systems, indicating that it is capable of detecting gross setup errors.

The feasibility of placing the IRRM on abdominal area for abdominalpelvic patients has been investigated. Maximum deviation of the system with respect to CIS was less than 6.0 mm, but reached 9.9 mm when compared with CBCT system. For the patients in this study, maximum daily CBCT shift along one direction was as high as 12.5 mm and patient breathing motion range reached 16.2 mm. These results have implication on the ability of the system to detect gross setup errors (when defined as unintended shifts greater than 10 mm). The system may generate frequent false alarms due to CBCT‐determined large shifts or breathing motion for patients with heavy breathing. To alleviate false alarms resulting from large CBCT shifts, the action level needs to be relaxed (for example, to 15.0 mm) along longitudinal direction. There are two ways to deal with false alarms resulting from large breathing motion. One is to place the CT BB (and IRRM) on patient chest area where less motion occurs. There are two issues with this method. Firstly, it may require an extra skin tattoo on the patient at the time of CT scan, which means extra work in the workflow and potential source of error in IRRM placement later at treatment. In addition, the chest area may not be included in the CT scan for all abdominalpelvic patients. Another way is to permanently transfer IRRM from patient skin to immobilization device in the first treatment fraction, as suggested by the authors. The drawback of this method is that, though accidental couch movement can be detected in real time, accidental patient motion cannot be monitored. A major advantage of this method is a streamlined, fully automated workflow. No therapist intervention is needed after the first treatment session.

### B. Importance of smooth clinic workflow

Significant emphasis was placed on achieving a smooth clinic workflow in the development of the system. The streamlined workflow was achieved through synchronization with the R&V system and mounting specially designed IRRMs directly on patient immobilization devices. The former allows for automatic loading of patient treatment information. The latter enables immediate and automatic tracking. These automations not only minimize therapists' workload, but also mitigate potential human errors in loading patient data or mounting the IRRM. A general concern with the use of reflective markers on patient skin for localization is the uncertainty associated with the placement of markers. Our method of permanently mounting IRRMs on patient immobilization devices minimizes such uncertainty.

In our opinion, a smooth clinic workflow is crucial for the successful employment of a safety system on a large scale. Nowadays, overall incident rate in radiotherapy is very low, and gross setup errors are even rarer, due to the widespread use of IGRT systems. A safety system must be efficient in detecting these rare errors. At our institution, the proposal of the safety system met initial resistance and reluctance from some therapists who claimed that the system would be redundant because of the use of daily CBCT imaging guidance. They were also concerned about the additional workload that would be added to an already busy schedule. However, the safety system was well adopted after deployment for two reasons. First, because of the smooth workflow, therapists gain extra confidence in patient setup at almost no expense. Second, the system caught several near‐misses in treatment rooms equipped with CBCT systems (discussed in the next section) and proved its effectiveness in ensuring patient safety.

### C. Error detection

In our experience, setup‐related accidents typically do not occur in routine treatments, but occur in unexpected and unusual circumstances. Our quality assurance and quality control systems are well designed to ensure patient safety in routine treatments. These systems may get bypassed under unexpected and unusual circumstances. For example, when treating patients having diseases with a large lateral offset (> 10 cm) from midline, collision between CBCT imaging source (or panel) and treatment couch (or patient) may be inevitable. In these cases, CBCT imaging can only be performed with a lateral couch offset less than planned by a few centimeters (e.g., 5 cm). After CBCT imaging, the difference can be accounted for through manual couch adjustment. This process needs additional human involvement and can be error‐prone. As another example, if a posterior beam goes through metal component in treatment couch top, an unconventional indexing position may be necessary as a result of shifting patient immobilization device longitudinally on the treatment table. If this information is not successfully communicated to substitute therapists in future treatment sessions, the patient can be set up to a wrong longitudinal position. Similarly, when treating patients on a different linac than planned (for example, due to machine being down) with different couch tops, different couch parameters are needed. The R&V system provides therapists the flexibility to override couch parameters at treatment console without double approval. This can be a source of error if the wrong couch parameters are used for initial patient setup. As a final example, image‐guided patient alignment using CBCT imaging or orthogonal filming may rely on image fusion of patient bony structures (e.g., spine and femur); anatomical similarities of these bony structures along the longitudinal direction may lead to misregistration and become a source of error. Manual registration or automatic software registration using a smaller region of interest than desired can significantly increase the likelihood of such errors.

Some errors resulting from these unusual circumstances can be detected before treatment starts, at the expense of treatment delay and unnecessary imaging exposure to the patient, while some errors (e.g., inadvertent couch shift after imaging and incorrect image registration) can go undetected and lead to treatment of wrong sites. Our PSS, providing independent and continuous patient position verification, can detect all these unusual errors when they occur, thus avoiding unnecessary imaging exposure and preventing severe treatment errors. The system is independent in the sense that it verifies patient setup by ensuring the position of the IRRM relative to treatment room isocenter. It is independent of treatment room, treatment machine, couch top design, immobilization device, or patient internal anatomy.

Since the deployment in April 2012 at our institution, the safety system has caught three near‐misses (potential gross setup errors). Without the safety system, two errors would be eventually caught by CBCT. In the first case, the indexing bar for a head and neck patient was placed at a wrong indexing hole (off by 7 cm) by a substitute therapist in CT scan, resulting in the wrong calculated linac couch positions. The second case was also related to our couch indexing system. In our institution, three of the four couch tops are the same; the fourth leads to approximately 14 cm difference in longitudinal couch parameter for the same treatment. When one machine breaks down and patients have to be temporarily treated on another machine, the difference is accounted for by creating setup beams with new couch parameters for the new machine. Instead of changing the couch parameters for all treatment beams, therapists are allowed to override them at the console after CBCT verification. In this case, the setup beams for the original machine were accidentally loaded for patient setup, causing 14 cm longitudinal offset in patient position. Both errors were caught by PSS before CBCT imaging, preventing patients from unnecessary CBCT exposure and potential treatment of the wrong sites. In the third case, a large (~1cm laterally) couch adjustment was needed after CBCT imaging. Due to concern of collision between gantry head and patient arms (lung patient with arms up position), treatment couch was arbitrarily moved laterally (~5cm) such that gantry could go to intended treatment position. However, the couch was not moved back and its parameters in R&V system were overridden. The offset in patient position was immediately caught by PSS at time of couch shift and caught therapists' attention before treatment. Without the PSS, this error could have gone undetected and resulted in treating a completely different site.

### D. Comparison with other tracking systems

Commercially available IGRT systems capable of continuous tracking such as ExacTrac Optical‐Tracking System, Frameless SonArray, and AlignRT have long been used for patient setup and monitoring, due to their high‐precision and continuous tracking ability.^(^ ^11^ ^,^ ^20^ ^,^ ^21^ ^)^ However, they all come with limitations compared to our system when trying to use them on a large scale as a general‐purpose patient setup system. Designed to monitor patient motion (both translation and rotation) during treatment, ExacTrac Optical‐Tracking System requires at least four IRRMs to be affixed on patient in each treatment session, which not only introduces significant workload to therapists but also may act as a source of error. Frameless SonArray system, which connects IRRMs in a fixed pattern to patient through a bite block, is only employed in the treatment of brain targets. Similarly, 3D surface imaging‐based systems AlignRT and C‐Rad Sentinel are not for general purpose, and predominantly utilized only in the treatment of brain, H&N, or breast patients. Most importantly, all these systems lack a streamlined workflow. For each treatment, therapists need to manually load the right patient under treatment and the correct treatment site, and provide couch angle for individual beams in the case of noncoplanar treatment (e.g., in AlignRT). Frequent therapist interventions not only disrupt clinic workflow, but may also introduce human errors, which discourages their large‐scale use as a patient safety system in a busy clinic.

### E. Limitations of the system

There are a few limitations of the system we need to consider. Instead of using commercially available spherical IRRMs, we designed flat‐surface, disposable IRRMs. These IRRMs are easier to fabricate and mount. But they are subject to visibility issue due to their small volume and flat surface. The view of the cameras can be obstructed by patient skin slope (in chest area) or when the IRRM surface is angled away from the cameras. For patients treated with face mask, we overcome the problem by affixing the IRRM over patient chin area and tilting the reflective surface toward the cameras. For other patients treated with body mold, the IRRM is shifted from patient skin onto a spot on the body mold. The flat surface of the IRRM is tilted to ensure visibility. Another benefit of doing so is the streamlined workflow, but the tradeoff is the loss of the ability in tracking patient motion throughout the treatment. To mount the IRRM on patient immobilization device instead of patient skin for tracking, reproducibility of the patient lying in the body mold is assumed. In our institution, it is ensured by aligning the two sets of skin tattoos (intended for correcting patient rotation) with marks on the body mold made at CT simulation.

To streamline clinic workflow, only one IRRM is used for each patient. The effects are two‐fold: (1) the system lacks submillimeter high precision, and (2) the system is not able to distinguish translational and rotational errors in patient setup. Neither of these affects the ability of the system to detect setup errors of more than 1 cm. Both translational error and rotational error lead to positional deviation of the IRRM relative to room isocenter, which triggers alarm in the system. These two types of errors can theoretically offset each other and mislead the system. However, the likelihood of such an event is low if the system is only used as a complementary patient safety system to safeguard patient treatment, but not used to guide patient setup.

## V. CONCLUSIONS

We have developed an efficient automatic general‐purpose PSS to prevent gross setup errors in radiotherapy. The system provides real‐time independent position verification for the treatment of all disease sites based on optical‐tracking technology, and is independent of treatment room, treatment machine, couch top design, immobilization device, or patient internal anatomy. The system has been well‐adopted into the use in a busy clinical environment because of its seamless workflow and effectiveness in catching setup errors. Due to its advantages of continuous tracking ability, no radiation dose, and fully automated clinic workflow, it can be an ideal complement to complicated IGRT systems in ensuring patient safety in radiotherapy.
